# Study of Arteriovenous Fistula Cases in a Tertiary Care Hospital: A Descriptive Cross-sectional Study

**DOI:** 10.31729/jnma.4957

**Published:** 2020-05

**Authors:** Robin Man Karmacharya, Satish Vaidya, Amit Kumar Singh, Sushil Dahal, Prasesh Dhakal, Niroj Bhandari, Sohail Bade, Prabha Shrestha, Pratima Thapa

**Affiliations:** 1 Department of Surgery, Kathmandu University Teaching Hospital, Dhulikhel, Kathmandu, Nepa

**Keywords:** *arteriovenous fistula*, *chronic kidney disease*, *Nepal*

## Abstract

**Introduction:**

Arteriovenous fistulas are a preferred choice for hemodialysis access in chronic kidney disease patients. There is increased adoption of arteriovenous fistula creation in Nepal. Our objective is to study various arteriovenous fistulas that have been created in our center.

**Methods:**

This is a descriptive cross-sectional study conducted in a tertiary care hospital including all cases of arteriovenous fistula creation from January 2018 to December 2019. We obtained the ethical clearance from the institutional review committee of Kathmandu University School of Medical sciences. Convenient sampling method was used. Detailed vascular mapping and color doppler ultrasonography was done in the bilateral upper limb as preoperative preparation and to choose a site for arteriovenous fistula creation. Data were entered into the Statistical Package for the Social Sciences version 20 for analysis.

**Results:**

Among 50 patients, the most common location was brachiobasilic 20 (40%) patients followed by brachiocephalic 18 (36%), radiocephalic 11 (22%), and arteriovenous graft between the brachial artery and axillary vein 1 (2%). The mean duration of hospital stay was 1.44 days. Three (6%) patients required re-intervention, all within 24 hours. Two (4%) patients had a failure of arteriovenous fistula requiring the creation of a new arteriovenous fistula.

**Conclusions:**

Brachiobasilic was the most common location for arteriovenous fistula creation. Reintervention was not common.

## INTRODUCTION

Chronic kidney disease (CKD) is a global public health problem with high mortality and morbidity, causing a significant financial burden to the patients and health systems.^1^,^2^ Nepal has a high burden of patients with chronic kidney disease requiring renal replacement therapy.^3^,^4^ Arteriovenous fistula is the preferred access for hemodialysis due to its longevity, low cost and less chance of infection.^5^ Interventions are needed to maintain its patency due to risk of maturation failure and thrombosis.^6^

There is little literature published regarding the practice and outcome of vascular access for hemodialysis and arteriovenous fistula creation in Nepal.^7^ Arteriovenous fistula was created in less than one-tenth of chronic kidney disease patients while the choice of site varied between the institution.^7^^10^

The main objective is to study arteriovenous fistulas created in Dhulikhel hospital with a specific focus on demographic parameters, sites, type of fistula, vessel mapping, and maturation information.

## METHODS

This is a descriptive cross-sectional study conducted in the department of cardiovascular and thoracic surgery, Dhulikhel hospital from January 2018 to December 2019. The ethical clearance from the institutional review committee of Kathmandu University School of Medical sciences was taken before conducting the study. This study includes in all the cases on whom arteriovenous fistula was created for hemodialysis access. A convenient sampling method. In the case of fistula could not be created in the primary incision site causing a newer incision in another site, the site where the fistula was created was taken for analysis. The sample size was calculated using the formula,

n= Z^2^ × p × q / e^2^

= (1.96)^2^ (0.05) × (1-0.05) / (0.07)^2^

= 37.24= 37

Where,

n= sample size
p= prevalence of absence of thrill= 5%
q= 1-p
e= margin of error i.e. 5%

Taking a non-response rate of 10%, the sample size would be 40. Although the calculated sample size is 40, 46 participants were included in the study. In preoperative preparation, detailed vascular mapping was done in both upper limbs to note the size, flow, and presence or absence of thrombus in the brachial artery, radial artery, and ulnar artery. Diameter, compressibility, presence or absence of thrombus in the forearm cephalic vein, forearm basilic vein, arm cephalic vein, and arm basilic vein was checked with the help of doppler ultrasonography. The Acuson P300 ultrasonography machine (Siemens corporation) was used with linear probes of 7.5 to 10 megahertz (MHz) for doppler ultrasonography. Based on the vascular mapping appropriate site for the creation of arteriovenous fistula was decided. After the procedure, analgesics and proper wound care was advised.^12^

The functionality of the arteriovenous fistula was assessed by the presence of a strong pulse, bruit of continuous systolic-diastolic nature, and thrill at the fistula site.^13^,^14^ If the size of the vein was more than 6 mm, 6 cm long, the flow rate of more than 600 ml/ minute and less than 6 mm from the skin, the patients were tried for hemodialysis from the created fistula.^15^

In the absence of clinical findings for fistula use, doppler ultrasonography was done for confirmation. The absence of adequate flow to use for dialysis was considered as a failure of fistula. Failure of the fistula was confirmed by examination and supporting imaging. If patients could not follow up, they were interviewed by telephone along with information on the radiological proof for the usability of fistula. The data were entered into the statistical package for the social sciences version 20 for analysis.

## RESULTS

A total of 50 patients had arteriovenous fistula creation, 31 (62%) were male and 19 (38%) were female. The mean age was 51.3 years (standard deviation 15.2, range 22-87). About 21 (42%) had both hypertension and diabetes and 17 (34%) had hypertension, and 10 (20%) had diabetes. In 34 (74%) cases, the fistula was made on the left side. The most common location was brachiobasilic done in 20 (40%) patients followed by brachiocephalic in 18 (36%) patients. Intraoperative change of surgical site was not done in any case. In 11 (22%) patients, an arteriovenous fistula was made in a radiocephalic location. In 1 (2%) case arteriovenous graft was kept between the brachial artery to the axillary vein (Table 1).

**Table 1:** Location of fistula.

Location of fistula	n (%)
Brachiobasilic	20 (40)
Brachiocephalic	18 (36)
Radiocephalic	11 (22)
Arteriovenous graft (brachial artery to axillary vein)	1 (2)

The mean duration of hospital stay was 1.44 days (standard deviation 1.3, range 0-8). The number of cases done as a daycare basis was 8 (16%). A maximum number of cases were discharged on the first day (46%) followed by the second day (28%) (Table 2).

**Table 2:** Mean duration of hospital stay.

Mean hospital stay	n (%)
0 (day care)	8 (16)
1	23 (46)
2	14 (28)
3	2 (4)
4	2 (4)
8	1 (2)

Regarding anesthesia, in 34 (68%) cases local anesthesia (with/without sedation) was used while in 16 (32%) cases regional block (brachial block) was given (with/without additional local anesthesia and/or sedation. There were 3 (6%) cases of re-intervention required all within 24 hours. The cause of re-intervention was postoperative bleeding. In two cases bleeding was from anastomosis site while in a case the bleeding was from the first branch of the vein in which the applied tie was displaced. During follow up an evaluation or telephone interview (who could not come for follow up) till following up of a minimum of six months, two (4%) patients had a failure of arteriovenous fistula requiring the creation of new arteriovenous fistula (done in other centers). In both cases however, dialysis was done (for two and four months) before the failure of the fistula.

## DISCUSSION

Chronic kidney disease patients requiring hemodialysis are increasing in Nepal, and arteriovenous fistula is the recommended method for long term vascular access.^16^ Elaborated practice and outcomes of arteriovenous fistula for hemodialysis were mentioned in very few literatures of Nepal.^7^ ^9^ Renal replacement and arteriovenous fistula creation were done more in males.^8^,^9^ Diabetes and hypertension were the major cause of chronic kidney disease as is present throughout the world.^17^

From our study, the most common location of fistula creation was brachiobasilic 20 (40%) followed by brachiocephalic 18 (36%) and radiocephalic 11 (22%). A study from Vietnam had radiocephalic arteriovenous fistula (Brescia-Cimino fistula) as the most common site (92%) followed by ulnar artery basilic vein (3%) with failure to mature of 7.4%.^18^ Most of our cases were referred cases with previous fistula failure.

In our study, fistula creation was more common on the left side. This is because it is always preferable to create in the non-dominant side as this will have lesser impact on day to day activities compared to the fistula created in dominant side.^19^ Arteriovenous fistulas aren't the first method followed for vascular access for hemodialysis in Nepal. Among 82 patients requiring vascular access arteriovenous fistula creation was done in six patients with the majority of the patient having temporary venous access through the femoral vein.^8^ Among 100 chronic renal failure patients only 11 patients had arteriovenous fistula created before initiation of hemodialysis.^9^ Two patients had complications of primary failure among 30 arteriovenous fistula patients with primary failure due to secondary infection and post-operative hematoma formation.^7^ Very few studies in Nepal have the evaluation of maturation in the arteriovenous fistula. Three-fourth of the fistula matured within 6 weeks among 59 patients on whom brachiobasilic fistula (BBF) was created.^20^ Two (4%) patients had a secondary failure of arteriovenous fistula requiring the creation of a new arteriovenous fistula. Large scale studies and studies predicting maturation of fistula will help us answer many of the questions.

## CONCLUSIONS

Arteriovenous fistulas for renal replacement were created more in males. Brachiobasilic was the most common location for arteriovenous fistula creation. Local anesthesia was used more than general anesthesia. Re-intervention wasn't common and was done for postoperative bleeding. Failure was uncommon up to six months past surgery. Studies to explore different aspects of AV fistula cases in our context will help know many unanswered queries in the local population.

## Conflicts of Interest:

**None.**
